# Disease and Disorders of Freshwater Unionid Mussels: A Brief Overview of Recent Studies

**DOI:** 10.3389/fphys.2016.00489

**Published:** 2016-11-01

**Authors:** Francesca Carella, Grazia Villari, Nicola Maio, Gionata De Vico

**Affiliations:** Department of Biology, University of Naples Federico IINaples, Italy

**Keywords:** Unionidae, freshwater mussels, animal disease, comparative pathology

## Abstract

The use of aquatic invertebrates in biomedical research and as environmental sentinels has dramatically grown in recent decades, with an increased need in understanding of comparative pathology. The Unionids freshwater mussels are a group of worldwide distributed bivalves residing small ditches and ponds, lakes, canals and rivers, often used as animal test in eco-toxicological studies. Once one of the most abundant bivalve molluscs in ancient rivers around the world, now many of them are declining in many countries and consequently are nearly extinct in many areas. The causes of this decline are not fully understood but alteration and degradation of the freshwater habitat seemed to play a central role. To date, link causality to the observed losses during episode of mussel die-offs has been more difficult to establish, and disease and pathogen presence have been scarcely considered. In this article we provide a brief overview of unionids freshwater mussel conservation status, also describing reported diseases and pathogens and illustrating a few relatively well-documented studies.

## Introduction

**Figure 1:**
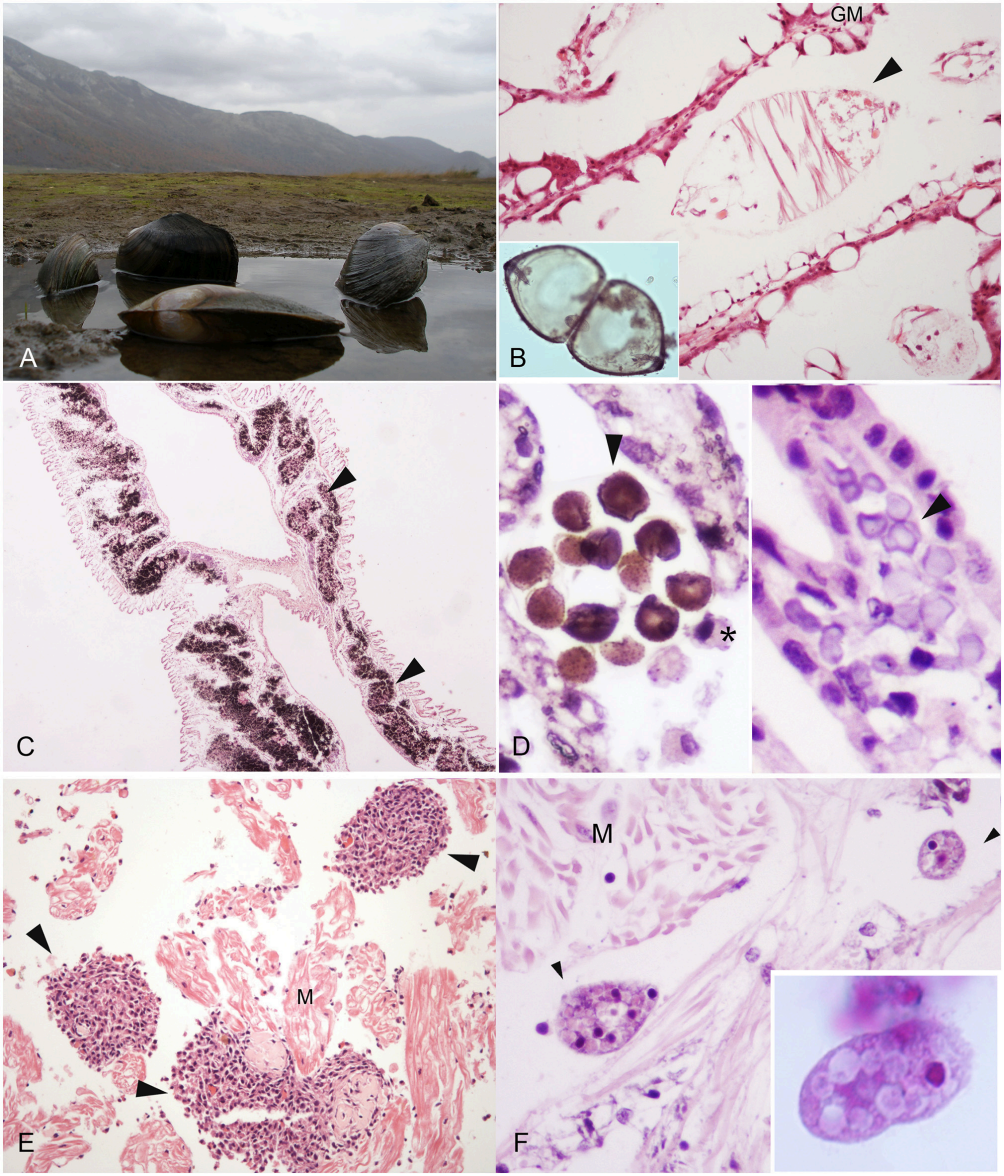
**Macro-microscopical observation of pathogen and disease in freshwater mussel species. (A)** *Anodonta cygnea* from Matese Lake (Caserta, Campania region) H&E; **(B)** Glochidium of *Anodonta woodiana* in the gill marsupium (GM), H&E, 40X; **(C)** Calcium Concretions (arrowheads) in the gills of *Unio pictorum* visible in black, Von Kossa Stain, 20X; **(D)** Calcium concretions in gills: Stain for Copper (Left), H&E (right) 40X; **(E)** Inflammatory nodulation (arrowheads) in heart muscle fibers (M) in *A. anatina* E&E, 40X. **(F)** *Conchophthirus* spp. (arrowheads) in the mantle of *Unio pictorum*, E&E, 100X. ^*^Haemocyte (M): muscle.

Invertebrate species represent a great percentage of animal diversity; however, they attract extremely minor research effort relative to vertebrates. Among them, non-marine molluscs (i.e., terrestrial and freshwater) are one of the most diverse and endangered animals with limited research specialists. In particular, freshwater mussels (Bivalvia: Unionoida) are a species-rich group of bivalves comprising about 900 nominal species in six families, including 300 species of Unionidae and 10 of Margaritiferidae, other than Hyriidae, Iridinidae, Mutelidae, Mycetopodidae with extant representatives ranging on all continents except Antarctica (Figure 1A). In particular, the characteristics that set the superfamily Unionoidea is the parental care of offspring until they are released as larvae, and the presence of parasitic larvae. Unionids adults are relatively sedentary, but in some species the larvae, the glochidia, are ectoparasite on fishes or amphibians, which provides a mechanism for dispersal. Concern about unionids populations has stimulated interest in the propagation of some species (Hanlon, 2003), but as for all cultured animals, there are numerous factors that affect the health of mussels reared in captivity (Jones et al., 2005). The potential for pathogens to kill or have sub-lethal effects on mussels in culture conditions has generally not been adequately evaluated. Here, we provide a brief overview of unionid freshwater bivalves considering animal conservation status; this is an attempt to describe the use of these organisms in eco-toxicological studies, also reporting diseases and pathogens of this group and illustrating a few relatively well-documented works.

## Conservation status

All too often, freshwater mussel populations are referred as objects of study for their noticeable decline in different part of the world. Once they were the most abundant bivalve mollusc in ancient rivers around the world, but now many of them are declining in all countries, with species nearly extinct in many areas. The causes of this decline are not fully understood, and have become alarmingly frequent and widespread over the past 20 years, with a number of short-term die-offs going largely unexplained but documented to some degree (Neves, 1987; Sparks et al., 1990; Fleming et al., 1995). Better documentation seems to be available for longer-term declines (Busby and Horak, 1993; Layzer et al., 1993; Williams et al., 1993). In many cases, alteration and degradation of the freshwater habitat like destruction of dams, modification of channels along with the introduction of non-indigenous mollusc, it seemed to play a central role (Williams et al., 1993; Saunders et al., 2002). A total of 200 unionoid species are on the IUCN Red List: 5 in Eurasia, 5 in Brazil, 1 in Australia, and the remaining 189 in the United States. Within the United States and Canada, 202 of the nearly 300 unionid species known are listed by the Natural Heritage Network as presumed extinct, possibly extinct, critically endangered, or vulnerable. In the United States alone, 37 species are presumed extinct or possibly extinct (Master et al., 2000). In Italy, five species of Unionoidae are present. *Anodonta cygnea* (Linnaeus, 1758), *Sinanodonta woodiana* (Lea, 1834), *Microcondylaea compressa* (Menke, 1830), *Unio elongatulus* (Pfeiffer, 1825) and *Margaritifera auricularia* (Spengler, 1793). Among them, *M. compressa* (= *M. bonellii*) is considered vulnerable in Italy and at the European level (Cuttelod et al., 2011; IUCN, 2015.4^1^) and is subject to management measures in European Union by the Annex V of the “Habitats Directive.” *U. elongatulus* (= *U. mancus*) is subject to management measures in European Union by the Annex V of the “Habitats Directive.” *Margaritifera auricularia* is currently listed as Critically Endangered at the European level (Cuttelod et al., 2011; IUCN, 2015.4). In the 1980's it was considered to be nearly extinct, then was object of an European Action Plan and conservation programs to determine suitable fish hosts and levels of tolerance to pollution.

The unionid group also include many alien species. According to the IUCN Red List of Threatened Species (Version 2015.4), the Chinese pond mussel *S. woodiana* is the largest freshwater bivalve (valve length of up to about 30 cm) and the fastest spreading, considered Invasive Alien Species in Europe (Cummings, 2011). This mollusc species originates from East and South-East Asia. The first record from Europe goes back to 1984 in Ungheria after its introduction in Danubian Basin (Petró, 1984) with some data for 1979 in Romania (Sárkány-Kiss, 1986; Frank et al., 1990). It was found in Italy for the first time between 1989 and 1996 (Manganelli et al., 1998; Watters, 1999), and in about 25 years, it formed colonies in 12 Italian regions (De Vico et al., 2007; Guarneri et al., 2014).

## Ecotoxicology

In the last years, evaluation of stressor impacts upon freshwater mussel communities has progressed to distinct phases of investigation. Among them, greater emphasis is placed on the enhancement of methodologies to guide sampling or monitoring programs toward well-suited objectives that offer greater perspective and resolution of mussel community or population status. The need for balanced guidance ranges from field to laboratory consideration of evaluative tests [American Society for Testing and Materials (ASTM), 2005], as well as surveying, monitoring and sampling acceptability [American Society for Testing and Materials (ASTM), 2001; Strayer and Smith, 2003]. These refined methods support different monitoring approaches taking into account water quality parameters, discharge limits, or even impacts of large-scale disturbance (Farris and Van Hassel, 2006; Collins et al., 2008; Grabarkiewicz and Davis, 2008).

Among the families of freshwater bivalves, there are different reasons for their use in eco-toxicological research. Firstly, the Dreissenidae, and to a lesser extent, the Sphaeriidae, have fulfilled the traditional role of eco-toxicological research organisms. These bivalve families have supported a large percentage of basic research on contaminant uptake, toxico-kinetics, and toxicity testing due in part to their broad distribution, abundance, high reproductive potential, and ease of collection and laboratory culture. The Unionidae, in particular, has emerged as a critical group for a consideration in the field of ecotoxicology over the past 20 years because of their high sensitivity to chemical exposures and a variety of other environmental stressors, on respect to other group of organisms (IUCN, 2015). Many of the current researchers conducted ecotoxicology studies on these organisms because of their sensitivity to a variety of environmental disturbance, ease of collection and handling, and/or the lack of reliable information to support conservation and management. About their employment to assess genotoxicity and their use as animal tests (Makala and Oikari, 1990; Mersch and Beauvais, 1997), Valenti et al. (2005) reported that the results provided by experimental toxicity tests are critical for their conservation. Numerous laboratory studies have been conducted on freshwater mussels in order to understand the role of contaminants in the decline of the populations (Valenti et al., 2005; Ingersoll et al., 2006). In these studies, early life stages of several mussels species other than juveniles and adults, have been considered (Cherry et al., 2002; Ingersoll et al., 2006). As a matter of fact, most Unionids have a complex reproductive cycle including an ectoparasitic stage on fish, the glochidia. After fertilization, eggs develop to larvae called glochidia that mature in specialized chambers, called marsupia, of the female's gills (Figure 1B). Glochidia are released into the water and attach to the gills or fins of a suitable host fish. After one to several weeks of the parasitic stage, glochidia transform to juvenile mussels, leave the fish host, and fall to the torrent or bottom of lakes starting the free-living juvenile stage. Many studies indicate that glochidia and juvenile mussels are more sensitive to some chemicals when compared to commonly tested aquatic organisms like cladoceran, amphipod and different fish species (Ingersoll et al., 2006; Bringolf et al., 2007a,b; Wang et al., 2007a,b; Gillis et al., 2008). The American Society for Testing and Materials (ASTM) (2006) provides a list of recommended test conditions for toxicity tests. Along with that, the development of physiological and biochemical tests or biomarkers of sub-lethal exposure are critical in assessing the condition of unionids. Toxicity end-points have also been established including survival, growth, bioaccumulation and behavior, along with numerous molecular biomarkers such as glycogen concentration, DNA strand breakage, cellulolytic enzyme activity, and AChE inhibition [American Society for Testing and Materials (ASTM), 2006; Kolarević et al., 2013]. Several textbooks and review articles have been written on the use of biomarkers in a wide range of aquatic organisms (McCarthy and Shugart, 1990; Van der Oost et al., 2003), many of these have application in unionids (Farris and Van Hassel, 2006). In particular, there have been studies that have assessed mussel sensitivity to a range of environmental contaminants including pesticides (Keller and Ruessler, 1997), ammonia (Augspurger et al., 2003; Newton and Bartsch, 2007), mercury (Valenti et al., 2005), cadmium (Markich et al., 2003; Wang et al., 2007b). However, for a given chemical, toxicity can vary by an order of magnitude among life stage and species (Cherry et al., 2002; Augspurger et al., 2003). About heavy metals and pollutants like polycyclic aromatic hydrocarbons (PAHs), are commonly released into the environment by anthropogenic activities. In mussels, study on bioaccumulation have been conducted, in some case concluding that some species, like *E. complanata*, can be an effective biomonitor of PAH and PCB concentrations in aquatic systems (Gewurtz et al., 2003). Moreover, when exposed to elevated metal concentrations in their environment, molluscs are known to accumulate metals to high levels in their tissues (Hemelraad et al., 1986; Couillard et al., 1993). Metal tolerance in such organisms involve the sequestration of metals in non-toxic forms. Among the available sequestration sites in the intracellular space, high affinity metal-binding proteins such as metallothioneins (MTs), lysosomes and granules, also called concretions, are reported (Mason and Jenkins, 1995). Metallothioneins are low molecular weight, cysteine-rich metal-binding proteins and biochemical indicator of pollutant exposure (Roesijadi, 1992). Gill metallothionein concentrations in the freshwater unionid bivalve *Pyganodon grandis* vary both spatially and temporally along cadmium gradients (Couillard et al., 1993). In addition to metallothionein, unionids also produce calcium concretions (Figures 1C,D), present at the level of connective tissue of the gills (Silverman et al., 1987), in the mantle (Adams et al., 1997) and in the digestive gland epithelia (Pynnonen et al., 1987). The calcium present in the concretions is generally associated to phosphate (Mason and Jenkins, 1995), present either as orthophosphate (PO4), pyrophosphate (P2O7) or their protonated forms (Jefree et al., 1993). These structures are a calcium reservoir, serving as a source of calcium for shell development (Silverman et al., 1985), but can also play a role in the detoxification of metals (Simkiss, 1981; Mason and Simkiss, 1982; Lautiè et al., 1988).

## Biological agents and diseases

An important step in comprehending freshwater mussel health status is to gain information about pathogens. Parasites and infectious agents with related lesions (i.e., inflammations and regressive phenomena) of this group is poorly described in literature. Among the reported pathogens, bacteria, protozoan and metazoan parasites like trematodes, nematodes, mites, and ciliates (*Conchophthirus* spp.), have the potential to decrease the fitness of the host unionid, but their role in diseases has not been well established (Table 1). About diagnostic procedures, techniques were described by Southwick and Loftus (2003). Moreover, Fuller (1974), McMahon and Bogan (2001), Smith (2001) and Grizzle and Brunner (2009), presented a brief review of the diseases of freshwater mussels. Presence of inflammatory capsules and infiltrates have been observed in *Anodonta woodiana* linked to bacterial infection (Carella et al., 2013a; Figure 1E).

**Table 1:** **Pathogens (virus, fungi, protozoa, and metazoa) described in Unionids**.

**Regnum**	**Phylum**	**Class**	**Species**	**Bivalve hosts**
Virus	*Arenavirus*		*Lea plague Virus (HcPV)*	*Hyriopsis cumingii*
Fungi	*Heterokonta*	Oomycota	*Oomycetes saprofites*	*Unio* spp.
Protozoa	*Ciliophora*		*Conchophthirus* spp.	*Elliptio complanata, Anodonta marginata, Anodonta implicata, Pyganodon cataracta, Lampsilis radiata, Lampsilis cariosa, Alasmidonta undulata, Anodonta cygnea*
			*Heterocinetopsis unionidarum*	*Pyganodon (= Anodonta) grandis, Lasmigona complanata*
			*Trichodina unionis*	*Anodonta cygnea, Unio* spp.
Metazoa	*Platelmintes*	Trematodes Digenea	*Aspidogaster conchicola*	*Indonaia caerulea, Corbicula striatella, Lamellidens corrianus*
			*Cotylaspis insignis*	
			*Cotylogaster occidentalis*	
			*Lophotaspis interiora*	
			*Bucephalus polymorphus*	*Unio pictorum, Dreissena* spp.
			*Rhipidocotyle* spp.	*Unio pictorum, A. anatina*
			*Polylekithum* spp.	*A. plicata*
		Nematoda	*Hysterothylacium* sp.	*Diplodon suavidicus*
	*Artropoda*	Copepods	*Paraergasilus rylovi*	*Anodonta piscinalis*
		Mites	*Unionicola* spp.	*Unio complanata, Unio gibbosus U. ligamentinus U. intermedia, A. fragilis, A. footiana, A. cataracta, Anodonta cygnea, A. anatina, Elliptio complanata*
			*Najadicola* spp.	

Evidence for viral diseases has been found in only one species of freshwater bivalve, a Chinese pearl mussel, *Hyriopsis cumingii*. The *Hyriopsis cumingii*, Lea virus disease, which is often referred to Lea plague disease (HCPD), was first reported in 1980s. Next reports used light and transmission electron microscopic (TEM) analysis of tissues from diseased bivalve mussels showed that the HCPD was associated with an arenavirus agent termed *Hyriopsis cumingii* Lea plague Virus (HcPV) (Zhang et al., 1986; Zhong et al., 2011).

Differently to marine bivalves, little is known about bacterial diseases of this group of molluscs. All the reports present in literature still are uncertain about their role as pathogens/symbionts. In general, the importance of bacteria as etiological agent of diseases in marine bivalves is mostly reported in intensively cultured species. Previous studies from Jenkinson and Ahlstedt (1987) reported die-offs of unionids from the Tennessee River in the years 1985-1986 and observed the presence of different species of bacteria in the connective tissue and in the digestive gland of affected mussels, but related to scarce haemocytosis.

About protozoan reports, in unionids the most common group reported belong to the genus *Conchophthirus* spp. (family Conchophthiridae). Species of this genus are only found in freshwater bivalves, and are among the most common organisms in this animal group. The body of these ciliates is flattened, elliptical in profile, with the mouth near the middle of the body (Fenchel, 1965; Antipa and Small, 1971a; Figure 1F). They have dense cilia over their entire surface and an average length of about 100 μm. *Conchophthirus* spp. move within the mantle cavity and are not firmly attached to the host. The reported species by Kelly (1899) are *Conchophthirus anodontae* and *Conchophthirus curtus* were of 30 of the 44 species of unionids examined from Illinois and Pennsylvania.

Antipa and Small (1971b) described the presence of the ciliate *Heterocinetopsis unionidarum* (*Ancistrocomidae*) in 2 of the 4 species of mussels examined in the one locality where it occurred. Parasitized unionids like *Anodonta grandis* and *Lasmigona complanata* didn't show specific harmful effects from this protozoa, present at the gills and palps. Other reported ciliates belong to the genus *Tricodina*. This genus and related genera (Peritrichia: Trichodinidae) include numerous species reported as fish and marine bivalve parasites, but a few species are found in unionids. *Trichodina unionis* is found in the mantle cavity of *Anodonta cygnea* and *Unio* spp. in Europe (Fenchel, 1965). Prevalence approaches 100% in some populations but with only about 10 per host. Diameter of *T. unionis* is about 70–100 μm (Raabe and Raabe, 1961; Fenchel, 1965). The most common location of this organism is on the labial palps, and less often the gills (Raabe and Raabe, 1961). *Trichodina* sp. was observed in unionids collected in Illinois (Antipa and Small, 1971b) and North Carolina (Chittick et al., 2001). Histological examination did not reveal lesions associated with *Trichodina* sp. (Chittick et al., 2001). Other ciliates of unionids are the scyphidiid peritrich *Mantoscyphidia* sp., and low numbers of a scuticociliatid ciliate on the gills of *Elliptio complanata* in North Carolina (Chittick et al., 2001).

Among Metazoan parasites, larvae of nematodes belong to the genus *Hysterothylacium* (Nematoda, Anisakidae) parasitizing the pericardial cavity of *Diplodon suavidicus* from the Amazon basin (Brazil) were reported by Lopes et al. (2011), but no lesions pathogen-specific are described.

On the other side, Trematodes of different families are reported in this group of animals as mussels result as intermediate hosts for digenean trematodes. The family of Aspidogastridae commonly parasitize freshwater mollusks. Four species of aspidogastrids have been reported: *Aspidogaster conchicola, Cotylaspis insignis, Cotylogaster occidentalis*, and *Lophotaspis interiora*. Two of these species, are among the most common symbionts of unionids, are widely distributed, and are found in several hosts in North America (Huehner, 1984; Hendrix et al., 1985). Bucephalid trematodes in unionids belong to the genus *Rhipidocotyle* spp., recently called *Bucephalus polymorphus* (Kelly, 1899; Yanovich and Stadnichenko, 1997). In *Unio pictorum*, Baturo (1977) found sporocysts of *Rhipidocotyle campanula* and provided a detailed description of the developmental stages of this parasite. In Europe two species of *Rhipidocotyle* in the unionid *Anodonta anatina* are reported: *Rhipidocotyle campanula* and *Rhipidocotyle fennica* (Gibson et al., 1992; Muller et al., 2015). In North America the *Rhipidocotyle* spp. identified as parasite unionids are *Rhipidocotyle septpapillata* (Kniskern, 1952) and *Rhipidocotyle papillosa* (Woodhead, 1930, 1936). The most serious effect of bucephalid trematodes is host sterility with gonadal tissues replaced by sporocysts also accompanied to follicle fibrosis. Additional lesions also can occur at kidney level (Kelly, 1899; Kniskern, 1952; Taskinen et al., 1997; Yanovich and Stadnichenko, 1997).

Moreover, water mites like *Unionicola* spp. (Hydrachnidia: Unionicolidae) commonly occur as symbionts of freshwater mussels. More than half of the described species are considered as symbionts and in 2013 Edwards and Vidrine published a book on the topic, with information on biogeography, classification, mussel-mite interactions, coevolution and phylogenetics. Three genera are known as symbionts of freshwater molluscs, like *Dockovdia, Najadicola*, and *Unionicola* and generally they live on the gills, mantle or foot of their hosts (Vidrine, 1996; Edwards and Vidrine, 2013).

## Neoplastic diseases

During the past 50 years a considerable literature has been published on spontaneous and experimentally-induced tumors in invertebrates. Invertebrate neoplasia have been described in different taxonomic groups, like sipunculids, annelids, ascidians, arthropods, insects and bivalve with economic interest (Scharrer and Lochhead, 1950; Rosenfield et al., 1994; De Vico and Carella, 2015). In particular, gonadal and haemic neoplasia of marine bivalves are the most common, and present the characteristics of malignant tumors (Carella et al., 2013b). Others, less frequent type, are the tumors arise from epithelia, muscle and connective tissue, mostly classified as benign, with neither invasive behavior nor mitotic figures. These neoplasms like polypoid growths of the foot, mantle and pericardium have been found repeatedly in Unionid molluscs (Pauley, 1967a,b; Sparks, 1985) like *Anodonta cygnea* (Williams, 1890; Collinge, 1891), *A. implicata* (Butros, 1948) and *A. californiensis* (Pauley, 1967b). A fibroma, lined by simple columnar ciliated epithelium, arising from the palp of the mussel *A. implicata*, have been reported. Williams (1890) reported a pedunculated tumor, composed of glandular and muscle cells while Collinge (1891) also observed two tumors from the same species from the same species of freshwater mussel, with no microscopical descriptions.

## Conclusion remarks

In recent years, considerable progress has been made in our understanding of aquatic animal disease (Cockerell and Patterson, 2005; Boorman, 2008). Lately, research studies on bivalve pathology and immunity has been growing, demonstrating that invertebrates are capable of mounting a wide and complex immune response, with discovered molecular mechanisms behind this diversity unraveled in several aquatic species (Song et al., 2010).

Study results indicates that pathogens and diseases have the potential to impact conservation of this endangered aquatic fauna (Williams et al., 1993; Starliper, 2011). Comparative and anatomic pathology are an important component in ecological risk assessment, considering physical and biological stressors, as well as chemical contaminants (USEPA, 1992). Pathologists, Ecologists, environmental experts and governing organizations at all levels must be educated regarding the value of anatomic pathology in holistic risk evaluation in aquatic animal conservation.

Additional research is also needed to determine whether other types of pathogens are present in this group of bivalves. This increased emphasis on non-mammalian models is likely to growth the use of aquatic species in risk assessment, further highlighting the need to ensure a strong, optimally trained workforce in aquatic pathology that will use a standard approach in disease diagnosis.

## Author contributions

FC, Pathology part. GV, University of Naples Federico II–Technical support. NM, University of Naples Federico II–Conservation status od freshwater mussel expert. GD, University of Naples Federico II–Pathology part.

### Conflict of interest statement

The authors declare that the research was conducted in the absence of any commercial or financial relationships that could be construed as a potential conflict of interest.

## Footnotes

^1^I.U.C.N. (2015). IUCN Red List of Threatened Species. Version 2015.4. Available on: www.iucnredlist.org. Downloaded on 30 May 2016.
